# The pivot point arginines identified in the β-pinwheel structure of C-terminal domain from Salmonella Typhi DNA Gyrase A subunit

**DOI:** 10.1038/s41598-020-64792-w

**Published:** 2020-05-08

**Authors:** Ekta Sachdeva, Gurpreet Kaur, Pragya Tiwari, Deepali Gupta, Tej P. Singh, Abdul S. Ethayathulla, Punit Kaur

**Affiliations:** Department of Biophysics, All India Institute of Medical Sciences, New Delhi, 110029 India

**Keywords:** Biochemistry, Biophysics, Drug discovery, Structural biology, Pathogenesis

## Abstract

The essentiality of DNA Gyrase in basic cellular processes in bacterial pathogens makes it an ideal drug target. Though the Gyrase has a conserved mechanism of action, the complete DNA wrapping and binding process is still unknown. In this study, we have identified six arginine residues R556, R612, R667, R716, R766, and R817 in the DNA GyraseA – C-terminal domain from *Salmonella enterica* serovar Typhi (StGyrA-CTD) to be essential for DNA wrapping and sliding by a sequence and structure analysis. Through site-directed mutagenesis and EMSA studies, we observed that the substitution of R667 (blade 3) and R716 (blade 4) in StGyrA-CTD led to loss of DNA binding. Whereas, upon mutation of residue R612 (blade2), R766 (blade5) and R817 (blade6) along with supporting residue R712 (blade 4) a decrease in binding affinity was seen. Our results indicate that R667 and R716 act as a pivot point in DNA wrapping and sliding during gyrase catalytic activity. In this study, we propose that the DNA wrapping mechanism commences with DNA binding at blade3 and blade4 followed by other blades to facilitate the DNA sliding during supercoiling activity. This study provides a better understanding of the DNA binding and wrapping mechanism of GyrA-CTD in DNA Gyrase.

## Introduction

Topoisomerases are responsible for maintaining the topological state of DNA in the bacterial cell by releasing the torsional stress created during replication, transcription, and recombination^1,2^. They are classified as Type I or II based on the number of strands nicked in the initial round of activity. DNA Gyrase, a Type II topoisomerase, relaxes the positive helical turns by introducing negative supercoil in the DNA and prevents the overwinding of the genome^3–5^. Topoisomerase IV, a paralogue of DNA Gyrase, is known to relax positive supercoils and resolve the concatmers^6,7^. Functionally, DNA Gyrase exists as a hetero-tetramer composed of two subunits, GyraseA and GyraseB, as an A_2_B_2_ complex. GyraseA is composed of two domains, a 59kDa N-terminal domain which possesses the breakage and religation activity^8^, and a 37kDa C-terminal domain responsible for DNA binding activity^9^. Similarly, GyraseB has a 47kDa N-terminal domain having ATPase activity^10^ and a 43kDa C-terminal domain that interacts with GyraseA and DNA^11^. The enzymatic activity of DNA Gyrase has been explained by two strand passage mechanism, where the first strand of DNA termed as G-segment binds to the Gyrase A C-terminal domain (GyrA-CTD) of the enzyme. The second DNA strand, called T-segment, gets captured upon ATP binding to the Gyrase B N-terminal domain (GyrB-NTD). Upon ATP hydrolysis, cleavage takes place in the G-segment which introduces a break for the passage of T-segment. Once the T-segment has passed through, re-ligation takes place in G-segment, thus changing the linking number by two. The DNA binding to the enzyme is initially mediated by GyrA-CTD by placing the two DNA segments close enough to prevent superhelicity^12,13^. During this catalytic mechanism, the DNA binding and sliding across the Gyrase subunits is controlled by the β-pinwheel GyrA-CTD.

Though the sequence identity for DNA GyraseA- C-terminal domain amongst the different bacterial species varies from 42% in *B.subtilis* to 90% in *E.coli*, the catalytic mechanism of action remains the same^14^. The variations in the enzyme activity and the DNA specificity across the various organisms have been attributed to both sequence and structural differences in the C-terminal domain of Gyrase A. To date, GyrA-CTD structures have been reported from spirochete *Borrelia burgdorferi*^15^*, Escherichia coli*^16^*, Mycobacterium tuberculosis*^17^ and *Xanthomonas campestris*^18^. The structural studies reveal that GyrA-CTD adopts a disc or spiral shape anti-parallel β-pinwheel fold composed of six blades^15,19^ except in *B*.*burgdorferi* CTD where it is a flat β-pinwheel conformation. Divergence in GyrA-CTD shape and activity is due to the presence or absence of certain features like GyrA box ^20–22^ and acidic C-terminal tail^23,24^. The essential functional role of GyrA-CTD β-pinwheel fold is to wrap DNA and present G-segment for enzymatic action. The GyrA-CTD domain can alone efficiently bind and wrap DNA of minimum length ~ 40 bp to further introduce negative supercoil^25^. GyraseA which lacks the C-terminal domain is incapable of introducing positive writhe, thereby, simulating the action of the conventional Topoisomerase IV, which is capable of relaxing positive supercoils and decatenation^26^.

Gyrase A possesses a unique Q(R/K)RGG(R/K)G motif known as GyrA box in the GyrA-CTD domain which interacts with the DNA during segment presentation. The conserved motif is located near blade1 and the fold of this region is yet to be resolved structurally^15–17^. The GyrA box is crucial for both DNA wrapping and supercoiling activity and the deletion of this motif results in complete loss of activity in GyrA^21^. A previous study has been conducted to identify crucial residues involved in DNA wrapping in GyrA-CTD^27^. This study based on the sequence alignment of GyrA-CTD from *M.tuberculosis* with *E.coli* and *B.burgdorferi* has identified Y577, D669, R691, and R745 in blade3 as the residues important for DNA binding in *M.tuberculosis*(*M.tb*). In the present work, we have identified the crucial DNA binding arginine residues involved in Gram negative bacteria using structural and biochemical studies of GyrA-CTD from *Salmonella enterica* serovar Typhi. We determined the first crystal structure of GyrA-CTD from *S*. *Typhi* (StGyrA-CTD) at 2.4 Å resolution. Based on the structural and sequence analysis of GyrA-CTD, we have identified 6 conserved arginine residues, one from each blade which plays a significant role in DNA binding and wrapping. Our studies pinpoint, R667 and R716 located in blade3 and blade4 respectively, as the central pivot point residues for DNA binding and wrapping along with the GyrA box.

## Results

### Structure determination of StGyrA-CTD

**Figure 1:**
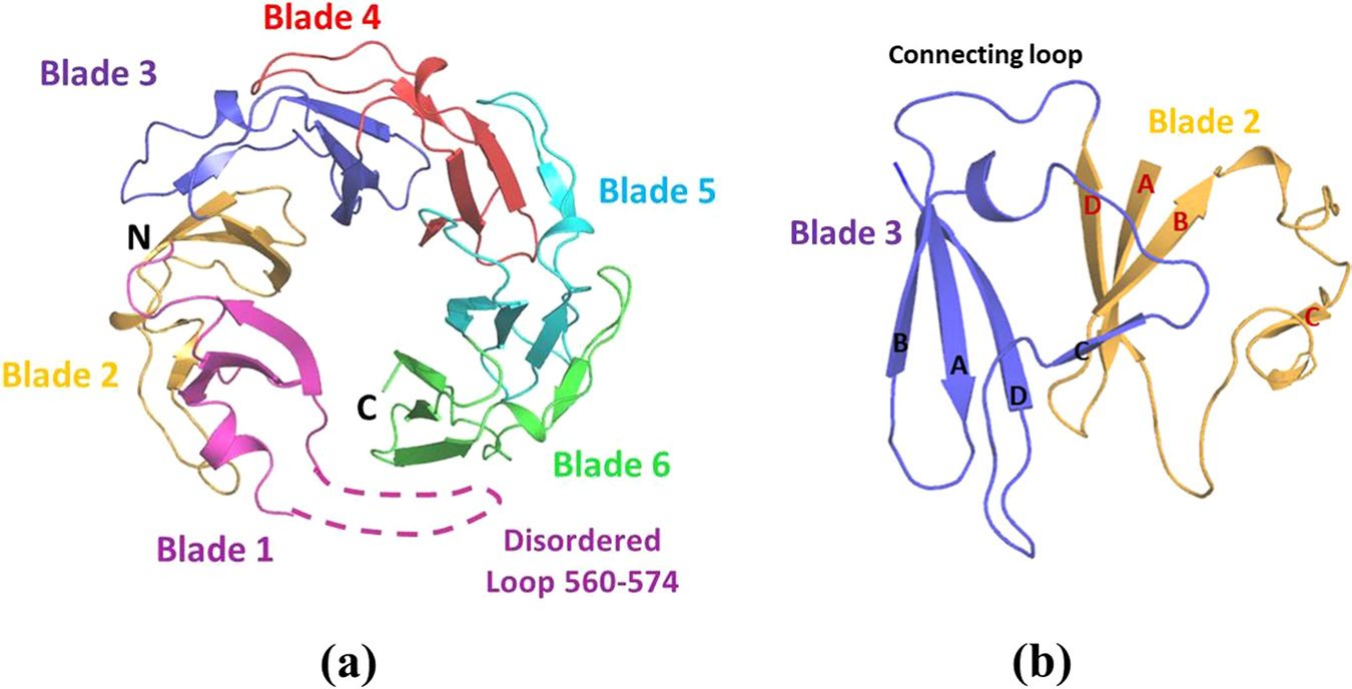
Structure of StGyrA-CTD. **(a) Cartoon representation of **StGyrA-CTD β-pinwheel structure with 6 blades. The dotted lines indicate the disordered GyrA-box (560–574) which could not be resolved in the crystal structure. **(b)** Blade 2 in yellow consists of 4 anti-parallel strands A, B, C, D linked to the next blade 3 in blue by a connecting loop.

The StGyrA-CTD was purified to homogeneity using Ni-NTA affinity chromatography (Fig. S1a) and size exclusion chromatography (Fig. S1b). Molecular weight calculated from the elution volume of the protein StGyrA-CTD indicates the protein to be a dimer in solution (Fig. S1c). The purified StGyrA-CTD was concentrated to 10 mg/ml and crystallized using hanging-drop vapor diffusion method. The crystal of StGyrA-CTD (residues 530–840) diffracted upto 2.4 Å resolution and belongs to the tetragonal space group P4_1_2_1_2 with unit cell dimensions a = b = 79.15, c = 87.36 Å **(**Table 1**)**. The StGyrA-CTD structure was solved by molecular replacement method using the *E.coli* GyrA-CTD (PDB ID 1ZI0^16^) as a template. StGyrA-CTD adopts the β-propeller fold comprising six repeated subdomains referred to as ‘blades’ to form a β-pinwheel structure (Fig. 1a). Each blade of the pinwheel constitutes a four-stranded antiparallel β-sheet structure (A, B, C, D) linked by a 15 residue extended loop (Fig. 1b). The GyrA box region from residues 560 to 574 is an extended β2-β3 loop which could not be resolved due to poor electron density map signifying that this region is completely disordered. This disordered region has not been observed in any structures of GyrA-CTD reported so far^15–18^. The deletion of the GyrA box hampers the negative supercoiling activity of Gyrase, thus emphasizing the significance of this loop^20–22^.

**Table 1:** Data collection and refinement statistics.

PDB Id	5ZTJ
**Data collection**	
Space group	P4_1_2_1_2
**Cell dimensions**	
a = b (Å), c (Å)	79.15,87.36
α = β = γ (°)	90.00
Resolution (Å)	58.66 (2.40)
Highest resolution range (Å)	2.64–2.40
*R*_sym_ or *R*_merge_ (%)	7.6 (58.3)
R_meas_ (%)	7.0 (12.52)
R_pim_(%)	2.9 (53.5)
I/σ(I)	14.89 (2.87)
CC(1/2)	99.8 (87.4)
Completeness (%)	99.9 (99.5)
Redundancy	5.62 (5.60)
**Refinement**	
Resolution range (Å)	47.1–2.40
Total number of measured reflections	116678
Number of unique reflections	20743
*R*_work_ */R*_free_ (%)	19.2/24.0
**No. of atoms**	
Protein atoms	2197
Water oxygen atoms	115
**B factors (Å**^**2**^**)**	
Wilson B-factor (Å^2^)	48.4
Avg. B-factor for Main chain atoms (Å^2^)	50.95
Avg. B-factor for water (Å^2^)	54.93
**R.m.s deviations**	
Bond lengths (Å)	0.005
Bond angles (°)	0.808

Values in parentheses correspond to the highest resolution shell 2.64-2.4 Å.

**Figure 2:**
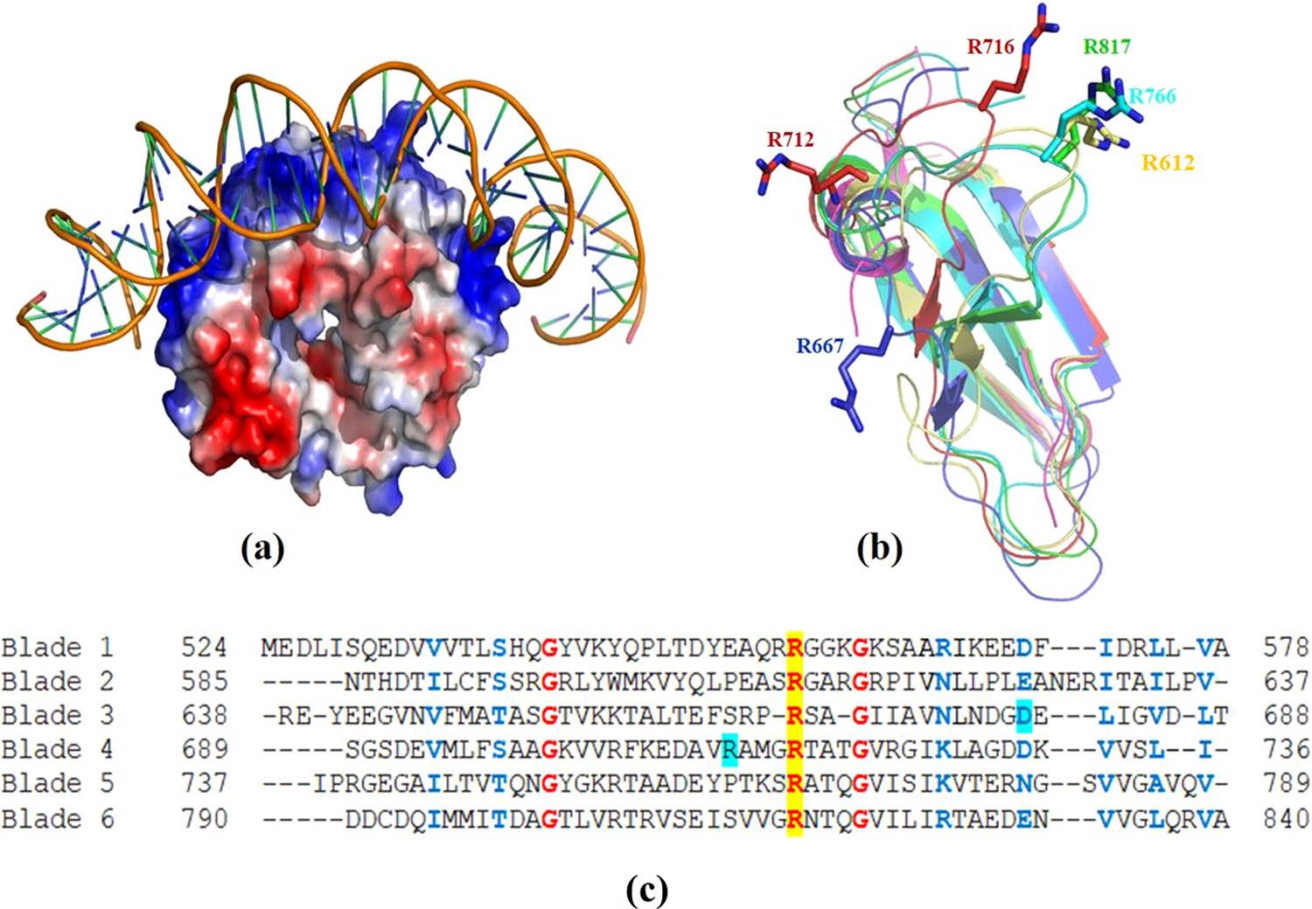
Selection of arginine residues. **(a)** Electrostatic surface potential of StGyrA-CTD prepared using Pymol indicates the charge distribution of basic residues around the perimeter of the beta-pin wheel structure docked with the 37 bp DNA. **(b)** Superimposition of all blades (blade1: magenta, blade2: yellow, blade3: blue, blade4: red, blade5: cyan, blade6: green) of StGyrA-CTD illustrating the conserved arginine residues. The position of arginine is almost conserved in blade2, 4, 5 and 6. **(c)** Sequence alignment of all six blades of StGyrA-CTD highlighting the conserved arginine residues in yellow and those identified from *M.tb* studies for mutational studies in cyan. The identical residues are in red whereas the similar residues are in blue.

GyrA-CTD forms a toroidal β-pinwheel structure^15^. It closely resembles the *E.coli* GyrA-CTD^16^ with an Cα root square mean deviation (r.m.s.d.) of 0.748 Å and is distinctly different from *B.burgdorferi* (Cα r.m.s.d. = 2.06Å) (Fig. S2). The structure is comparable with other homologs such as *M.tuberculosis* and Topoisomerase IV with r.m.s.d of 1.08 Å and 1.26 Å respectively for the Cα atoms. The electrostatic surface potential of StGyrA-CTD revealed that the positively charged residues are distributed uniformly around the perimeter of the β-pinwheel across all blades (Fig. 2a). The basic charge on the protein surface is largely contributed by the arginine and lysine residues present on the extended β2-β3 loops of all blades. This indicates that the DNA wraps around the perimeter of the StGyrA-CTD β-pinwheel structure during G-segment presentation. The superimposition of the individual blades of StGyrA-CTD with r.m.s.d of 1.52 Å for the Cα atoms suggested that the overall fold adopted by each blade is comparable (Fig. 2b). The sequence alignment of the individual blades depicted that the distribution and position of the charged amino acid residues in each blade in StGyrA-CTD is significantly conserved. It was observed that arginine residues, R556 (blade1), R612 (blade2), R667 (blade3), R716 (blade4), R766 (blade5), and R817 (blade6) could be identified as the potential DNA binding site in each blade (Fig. 2c).

**Figure 3:**
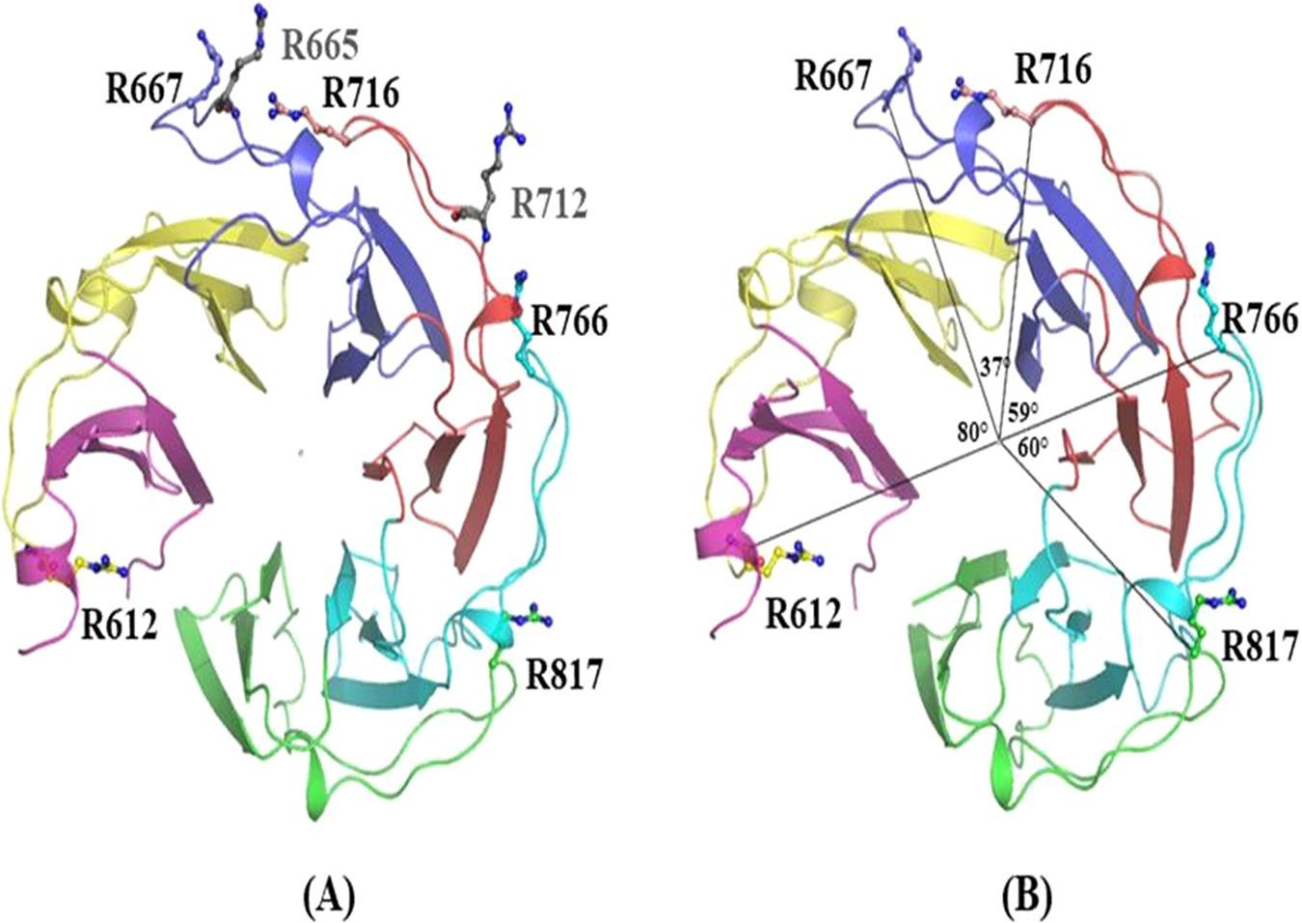
Interacting arginine residues identified in the structure of StGyrA-CTD. **(a)** Conserved arginine residue in each blade along with the supporting residues in blade3 and blade4 shown in grey **(b)** Angular displacement of arginine around the β-pin wheel. The angle between arginines from two blades concerning the centroid of protein is depicted. The R667 and R716 are pivot residues, R712 and R665 are pivot supporting residues and R612, R766, R817 are sliding residues.

The StGyrA-CTD structure revealed that the identified arginine residues were majorly distributed on the surface of the protein (Fig. 3a) and were conserved across species (Fig. S3). Further, to understand the significance of the conservation of an arginine position in each blade, the center of mass (CM) of the β-pinwheel structure was determined. Additionally, the angular distribution of each arginine residue from the center of mass was calculated. The angle formed between the Cα atom of arginine residue from one blade and the Cα atom of arginine residue in the consecutive blade to the center of mass of StGyrA-CTD was then measured (Fig. 3b). The angle between the blade1 and blade2 for R592 could not be calculated as the region, GyrA box, was completely disordered and could not be resolved in the crystal structure.

The angle distribution exhibited the maximum angle of 80.12° between blade2 - blade3 and angle of 60.64° between blade5 - blade6 for the arginine residues located at the edge of the β-pinwheel (Fig. 3) (Table 2**)**. The angular distribution of arginine residues located in blade3 - blade4 (34°) and blade4 - blade5 (59.8°) revealed a comparative decrease in the angles **(**Table 2**)**. The angular calculations suggest that the decline in the angle between residue R667 in blade3 and R716 in blade4 at the center of the StGyrA-CTD β-pinwheel acts as a pivot point for clasping the DNA. The observed increase in the angle in the adjacent blades indicates that these residues might contribute to the bending of the flanked DNA towards the surface wall formed by the β-pinwheel structure.

**Table 2:** Angular displacement between the centroid of protein and arginine of the consecutive blades.

CαArg – CM - CαArg	Angular displacement(°)
R612 (blade2) – CM - R667 (blade3)	80.12
R667 (blade3) – CM - R716 (blade4)	37.08
R716 (blade4) – CM - R766 (blade5)	59.80
R766 (blade5) – CM - R817 (blade6)	60.64

where CM – Center of Mass of StGyrA-CTD domain.

Based on sequence and structure analysis of the six blades, the conserved arginine residues R612 (blade2), R667 (blade3), R716 (blade4), R766 (blade5), and R817 (blade6) were chosen for biochemical and functional studies. Apart from these arginine residues, other residues D692, R712 and R766 corresponding to residues D699, R691and R745 respectively identified for DNA binding in *M.tb* GyrA-CTD[27] were also included for the DNA binding study. All the chosen residues were mutated to alanine by site-directed mutagenesis and all mutant StGyrA-CTD were purified using Ni-NTA and gel filtration chromatography with the same protocol as followed for the wild type.

### Electrophoretic-mobility shift assay (EMSA)

**Figure 4:**
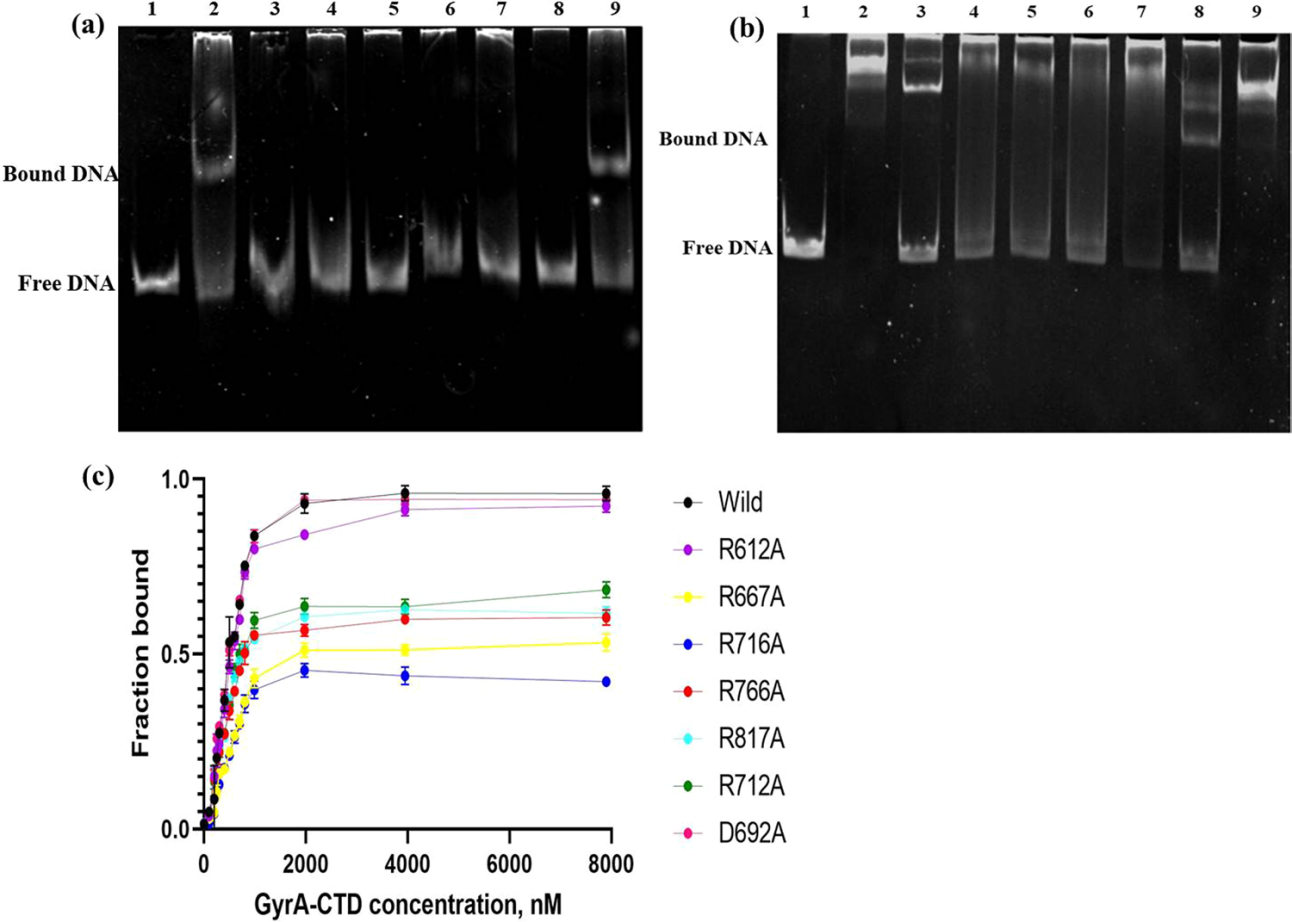
Gel retardation assay of wtStGyrA-CTD and mutants with **(a)** 37 bp and **(b)** 162 bp DNA. For 37 bp, a 5:1 ratio was used and for 162 bp 20:1 ratio was taken. In both assays, Lane1:free DNA, Lane 2: wild, Lane3: R612A, Lane4: R667A, Lane5: R712A, Lane6: R716A, Lane7: R766A, Lane8: R817A and Lane9: D692A. **(c)** Represents the DNA binding studies of StGyrA-CTD with FAM labelled 37 bp DNA (20 nM) by fluorescent binding assay.

The effect of a single point mutation in the StGyrA-CTD upon DNA binding was studied by the mobility shift of the DNA-protein complex. The unbound free DNA could be distinctly identified in the EMSA. The assay was performed using 37 bp and 162 bp DNA sequences reported in previous studies^17,24,28^. The EMSA illustrated that wild type StGyrA-CTD binds efficiently with both 37 bp and 162 bp DNA (Figs. 4a,b), signifying that the purified protein is properly folded. DNA binding was not observed in the studies with all StGyrA-CTD mutants (R612A, R667A, R712A, R716A, R766A, and R817A), except the mutant D692A, with 37 bp DNA implying their essential role in DNA binding (Fig. 4a**)**. In contrast, with 162 bp DNA, the mutants, R612A (blade2), R712A, R766A (blade5), and R817A (blade6) revealed partial binding with the presence of both protein-DNA complex and free DNA indicating weaker DNA binding (Fig. 4b). However, the mutants, R667A (blade3) and R716A (blade4) did not demonstrate any DNA binding even with 162 bp DNA establishing the importance of these residues in DNA binding and wrapping by gyrase. Surprisingly, the mutant D692A did not suggest a loss in DNA binding and behaved like the wild type protein indicating that the residue D692 has no role in interaction with DNA. This is in contrast to MtbGyrA-CTD where DNA binding was not observed for the D692A mutant^27^.

### Fluorescence binding assay

The DNA binding affinity of wild type and mutant StGyrA-CTD were measured using FAM-labeled 37 bp DNA by fluorescence binding assay with constant DNA and increasing protein concentration. With 37 bp DNA, the wild type StGyrA-CTD gave a K_D_ value of 122 nM (Fig. 4c). The K_D_ values for the mutants, R667A (blade3), and R716A (blade4) were 277 nM and 262 nM respectively. This demonstrated an approximately two-fold decrease in binding affinity in comparison to wild type. Barring the mutant, D692A, a similar trend was observed for other mutants with K_D_ value ranging from 220 to 250 nM. The binding affinity for the mutant D692A with K_D_ = 129.3 nM was comparable to the wild type StGyrA-CTD. This corroborated the EMSA study that the residue D692 was not involved in DNA interaction **(**Table 3**)**. The binding studies indicate that the identified residues in StGyrA-CTD except for D692 are crucial for DNA binding.

**Table 3:** Dissociation constant (K_D_) of wild and mutant StGyrA-CTD.

S.No	StGyrA-CTD	Dissociation constantK_D_ (nM)
1.	Wild type	122.5
2.	R612A (blade2)	134.9
3.	R667A (blade3)	262.9
4.	R716A (blade4)	277.1
5.	R766A (blade5)	221.4
6.	R817A (blade6)	204.3
7.	D692A	129.3
8.	R712A	231.3

### Protein-DNA Docking

**Figure 5:**
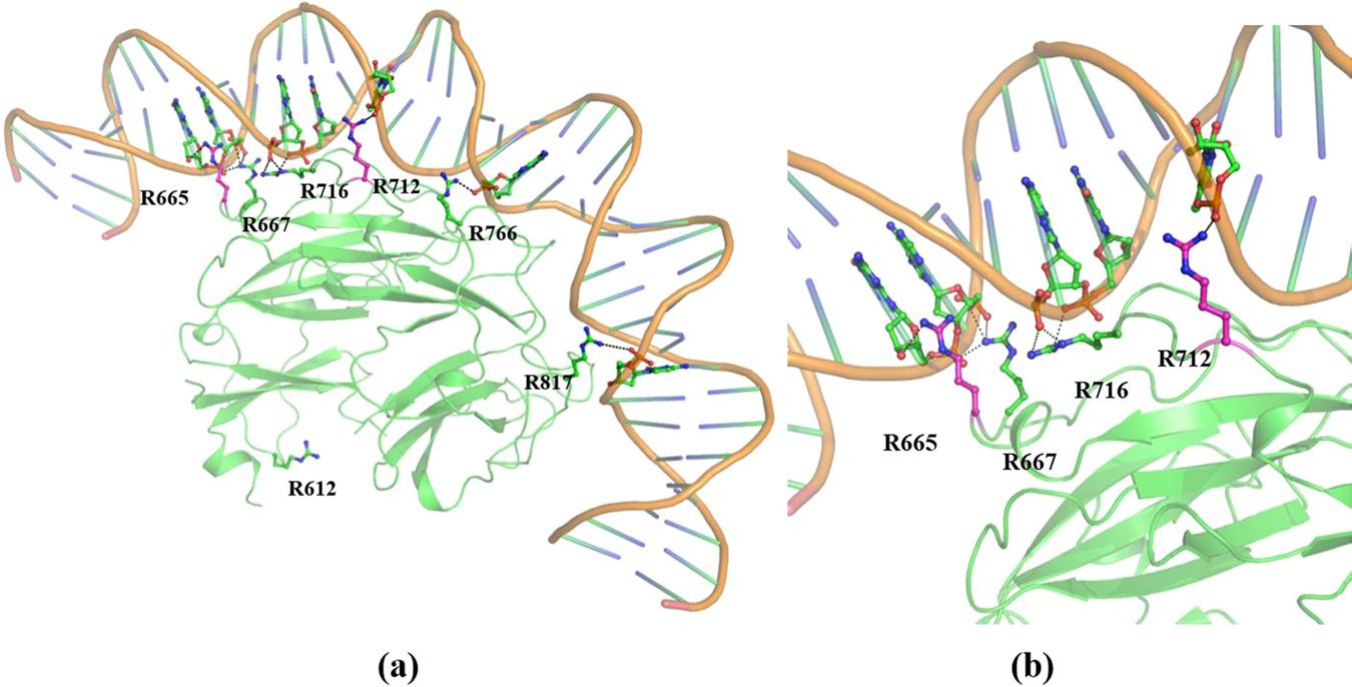
Docked position of StGyrA-CTD with DNA **(a)** Represents the StGyrA-CTD docked with 37 bp DNA using HADDOCK server. The residues involved in the DNA interactions are shown in sticks. **(b)** Close-in view of major interactions made by pivot residues in blade3 and blade4 represented in green alongwith supporting residues in pink.

The residues identified based on EMSA and fluorescent binding were further assessed for their interaction with DNA. Hence, molecular docking was performed using the HADDOCK server for both StGyrA-CTD wild type protein and the mutants with 37 bp DNA to understand the interactions occurring between the protein-DNA complexes. The docking studies were performed with ambiguous interaction restraints (AIRs) wherein 21 amino acid residues (R596, R598, K603, R612, R615, R617, R638, K656, K657, R665, R667, R705, K707, R712, R716, R722, K725, R630, R739, K754, and R755) were defined as protein-DNA interface active residues. HADDOCK score was calculated based on the summation of electrostatic, van der Waals interactions, desolvation and restraint violation energies. Based on the ranking in the structure cluster, the StGyrA-CTD - DNA complex with the lowest energy and higher binding affinity with a HADDOCK score of -162.8 kcal/mol was selected for structure analysis. The wild type StGyrA CTD - DNA complex revealed that the residues involved in the interaction with DNA were predominantly arginine residues. It was observed that one arginine residue from each blade (R667, R712, R716, R766, R817) directly contributed to the complex formation. The majority of the interactions involved in the protein-DNA complexation were hydrogen-bonded interactions formed between the arginine side chain and the base pairs primarily present in the major groove of the DNA backbone (Fig. 5a,b). A protocol similar to the wild type was used to perform docking with the 37 bp DNA with StGyrA-CTD mutants. The HADDOCK scores exhibited that majority of the mutant - DNA complexes displayed lesser affinity in comparison to wild type indicating the role of these residues in DNA interaction **(**Table 4**)**. Particularly, a lower Haddock score was observed for the mutants R667A (blade3), R716A (blade4), R766A (blade5), and R817A (blade6), suggesting a comparatively weaker binding with DNA than the wild type.

**Table 4:** Analysis of StGyrA‐DNA interaction obtained from HADDOCK calculation.

S. No	StGyrA-CTD	Haddock score	van der Waals energy		Electro-static energy	Desolvation energy	Restraints violation energy	Buried Surface Area
1.	Wild type	−164.9	−90.8	−789.8		37.4	464.7	2717.2
2.	R612A (blade2)	−163.4	−66.2	−849.0		34.3	383.3	2217.1
3.	R667A (blade3)	−158.3	−60.9	−889.2		31.7	488.0	2176.0
4.	R716A (blade4)	−145.8	−58.3	−817.8		33.0	430.9	2064.8
5.	R766A (blade5)	−153.3	−79.5	−760.3		34.6	436.8	2373.3
6.	R817A (blade6)	−156.8	−60.9	−889.2		31.7	488.0	2176.0
7.	D692A	−176.8	−79.9	−885.1		34.0	461.9	2441.1
8.	R712A	−155.0	−69.5	−744.1		24.3	389.8	2174.9

Two mutants, R612A (blade2) and D692A indicated considerable binding similar to the wild type protein denoting that these residues were not directly involved in hydrogen-bonded interactions with DNA. However, the R712A mutant (blade3) which is not directly involved in interactions with DNA presented a low negative score. The residue, R712, is significant as it provides a supporting role by maintaining the residues, R667 and R716, in the proper orientation for DNA binding. The protein - DNA docking studies thus support the biochemical data and establish the crucial role of residues R667 in blade3 and R716 in blade4 in DNA binding. The study further illustrates that the substitution of these two arginine residues will lead to a complete loss of DNA binding and mutations at the adjacent blades will compromise their affinity for DNA. The buried surface area (BSA) calculations for the wild type- and mutant - DNA complexes displayed the wild StGyrA-CTD to possess a higher BSA value of 2382.4 ± 147 Å^2^ as compared to the mutants **(**Table 4**)**.

## Discussion

DNA Gyrase, a type II DNA topoisomerase, is an essential enzyme that introduces positive and negative supercoiling to facilitate DNA replication in bacterial pathogens. The DNA Gyrase hetero-tetramer comprising GyrA and GyrB subunits is responsible for the overall supercoiling activity. The DNA wrapping during replication is completely modulated by GyrA-CTD domain. The wrapping of DNA is a crucial process for the cleavage of DNA G-segment and the passage of T-segment in the breakage reunion complex. It is also crucial for introducing positive or negative supercoiling in DNA. The DNA wrapping involves non-specific interactions formed between GyrA-CTD and DNA. However, the residues involved in these interactions are conserved in the blades of the β-pinwheel structure with the GyrA box determining the geometry of the bound DNA. DNA binding is initiated with the interaction of the GyrA box motif in blade1. This is followed by wrapping and bending of DNA around the surface of GyrA-CTD. The β-pinwheel structure of StGyrA-CTD presents uniform displacement of each blade from the center of the axis. Moreover, it acts as a pulley wheel with the major interactions explicitly formed by specific residues at definitive positions in the blades. In StGyrA-CTD, the basic residues are distributed evenly across the surface of the β-pinwheel structure. The arginine residues, R667, R712, R716, R766 and R817 located across the blades are the crucial amino acid residues required for DNA binding (Fig. 5a). The identified residues are distributed at a specific angle from the centroid in the StGyrA-CTD β-pinwheel pulley. The angle formed between two consecutive arginines from their respective blades and the centroid of protein provides a direct measurement of the angular displacement necessary for bending and twisting of DNA by StGyrA-CTD. The required inclination gradually increases from the side of the StGyrA-CTD towards the central arc **(**Table 2**)** and decreases from the center of the arc. The residues R667 and R716 present on blade3 and blade4 respectively form the pivot center. The mutation of these pivot center residues prevents the protein - DNA complex formation as observed in the EMSA studies with both 37 bp and 162 bp DNA sequences with a drastic decrease in DNA binding affinity (K_D_ = 277.1 nM for blade3 and K_D_ = 268.3 nM for blade4) in comparison to similarly positioned residues located in the other blades **(**Table 3). The detailed analysis of the StGyrA-CTD structure docked with DNA illustrated that the residues R667 and R716 form direct hydrogen-bonded interactions with DNA bases. The arginine residues, R665 and R712, situated two to four residues away but located in the same blade supplement the support to the pivot binding point (Fig. 5b). We hypothesize that the residues situated on the two blades, blade3 and blade4, act as a pivotal point with other residues, R612 (blade2), R766 (blade5) and R817 (blade6), to facilitate DNA passage through the fixed pivot point during supercoiling activity. The EMSA study corroborates our hypothesis, where the 162 bp DNA displayed incomplete binding with the mutants R612 (blade2) R766 (blade5) and R817 (blade6). This suggests that the primary function of these residues is to hold the DNA around the pulley like β-pinwheel and facilitate the DNA sliding during catalytic mechanism.

**Figure 6:**
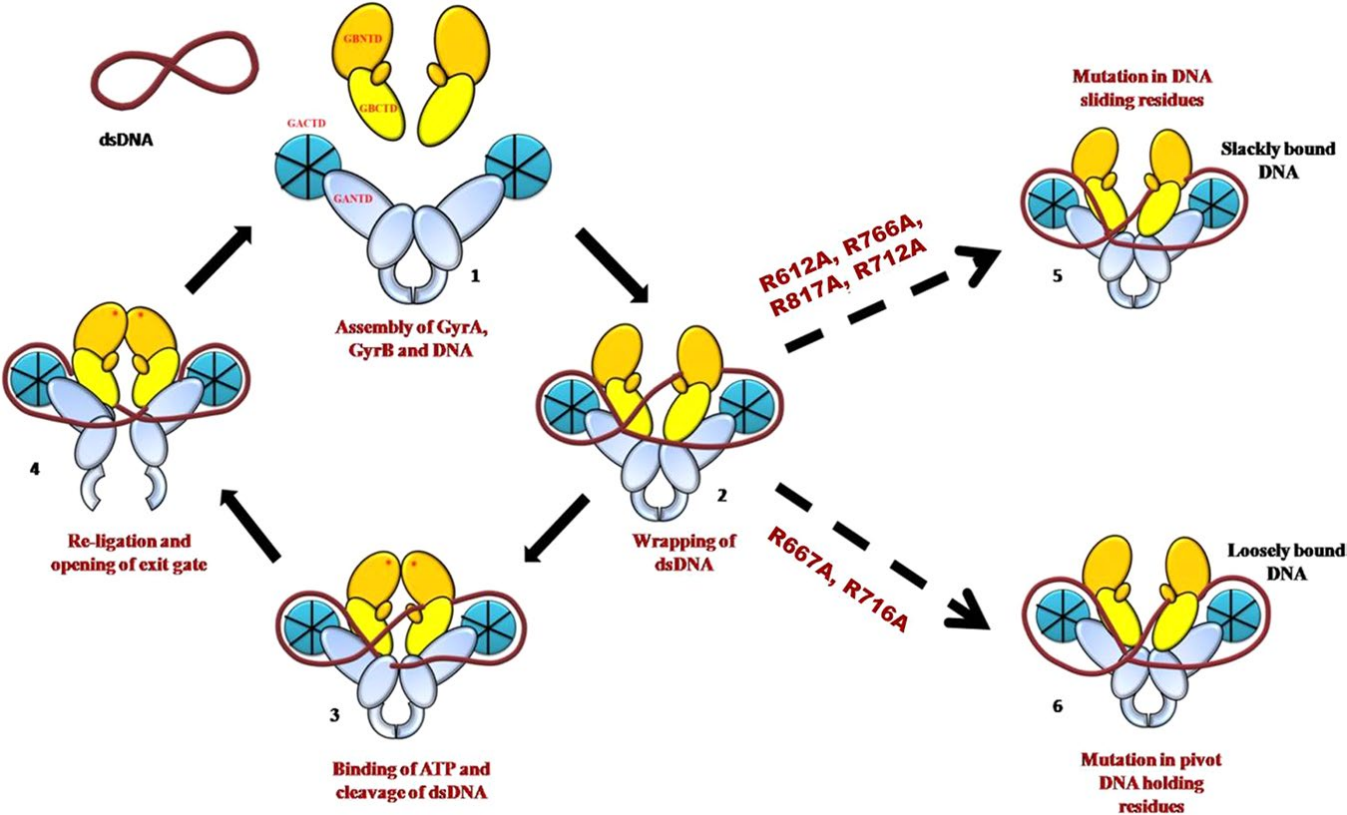
Schematic representation of DNA Gyrase catalytic mechanism. **1**. GyrA, GyrB associate with DNA to form holoenzyme **2**. dsDNA gets wrapped around the StGyrA-CTD presenting G segment for cleavage. **3**. ATP binding induces closure of ATP gate with DNA cleavage to form ssDNA by central core domain formed by GyrA-NTD and GyrB-CTD **4**. Nicked DNA gets resealed back to dsDNA with the opening of the exit gate. **5**. Mutation at position R612 (blade2), R766 (blade5), R817 (blade6) referred to as sliding residues and blade3 (712) referred to as supporting residues leads to the incomplete wrapping of DNA. **6**. Mutation of StGyrA-CTD at position R667 and R716 in blade3 and blade4 respectively referred to as DNA holding residues completely hampers its ability to wrap around the DNA.

Based on our study, we propose a DNA wrapping mechanism where the DNA binding in gyrase involves the association of GyraseA and GyraseB subunits with the DNA wrapped around StGyrA-CTD for the presentation of G-segment for activity. The wrapping induces ~150-degree bending of DNA G-segment facilitated by GyrA box and conserved arginine residues in StGyrA-CTD. Binding of ATP in the GyrB-NTD domain presents the DNA T-segment within the breakage reunion core complex, thus permitting the tight coupling of ATPase with DNA wrapping. At this juncture, the DNA wrapping by StGyrA-CTD is an important step for the activity of DNA gyrase. We propose that the DNA wrapping mechanism in gyrase acts like a revolving pulley with DNA hinged at a specific point to allow free movement along the sides of the pulley. The residues R667 and R716 in blade3 and blade4 respectively, act as a pivot point to hold the DNA in an appropriate orientation for strand passage. Once the DNA break is introduced at the catalytic subunit, StGyrA-CTD enables the smooth movement of DNA across gyrase protein. This is facilitated by the pivot point in StGyrA-CTD along with residues R612 (blade2), R766 (blade5) and R817 (blade6) in other blades for T-segment strand passage. Once the strand passage is accomplished, the linking number of DNA is altered, and the enzyme is ready for another cycle. A futile cycle may result due to incomplete wrapping around the StGyrA-CTD or inefficient movement of DNA during strand passage. Mutation of the pivot binding residues in blade3 and blade4 will abolish DNA binding, whereas, mutation of residues in other blades will cause DNA slack from the side of StGyrA-CTD with reduced efficiency of strand passage mechanism (Fig. 6). We propose that the residues identified in our study are not only vital for DNA binding but might play a key role in DNA supercoiling and relaxation activity by Gyrase.

In conclusion, we hypothesize that the mechanism of DNA wrapping involves the initial binding of DNA to blade3 and blade4.These two blades act as a pivot point and the other blades contribute to facilitate the DNA to move smoothly around the pivot point during supercoiling activity. Complete wrapping of DNA around StGyrA-CTD causes conformational changes resulting in the closure of the DNA gate after ATP binding. In this course of events, the DNA wrapped around StGyrA-CTD pulley is held by the pivot point formed by blade3 and blade4. For a cleavage reaction to occur, DNA passage is required at the interface of the central core region. The pivot handle point in the Gyrase CTD pulley enables the smooth passage of DNA allowing complete wrapping and efficient supercoiling activity. However, the proposed mechanism needs further study to provide a better understanding of these residues in DNA supercoiling and relaxation activities of DNA Gyrase.

## Materials and method

### Cloning of wtStGyrA-CTD and mutants

Truncated GyrA-CTD (residues 530–840) named as StGyrA-CTD was amplified from the gyrA gene of *Salmonella enterica* serovar Typhi (CT18 strain, Gene ID - 1250191) using a specific primer (forward, 5’-CATG**CCATGG**GCGAAGATCTGATTAGCCAGGAA-3’; reverse 5’- CCG**CTCGAG**GCCGTCGATAGC

GTCGAG-3’) having Nco1 and Xho1 restriction sites respectively. The amplified product was cloned into the pET-28a (+) vector with C- terminal 6x His tag. Residues selected for binding studies were mutated to alanine using *in vitro* site-directed mutagenesis where the whole plasmid was PCR amplified using mutagenic primers, treated with Dpn1 and sequenced to confirm the presence of a mutation.

### Protein purification

Wild type StGyrA-CTD and all its mutants were overexpressed in *E. coli* BL21-CodonPlus (DE3)-Rosetta cells by inducing the 0.5 O.D cells with 0.5 mM IPTG for 16 hours at 25 °C. The induced cells were harvested by centrifugation at 6000 rpm for 10 minutes at 4 °C and resuspended in lysis buffer (25 mM Tris pH 7.5, 0.5 M NaCl, 5% glycerol). The cells were disrupted by ultrasonication and the suspension was loaded onto the Ni-NTA column equilibrated with lysis buffer. Two-column volume wash was given using washing buffer (25 mM Tris pH 7.5, 0.2 M NaCl, 5% glycerol, 5 mM imidazole) and then the protein was eluted using the elution buffer (25 mM Tris pH 7.5, 0.2 M NaCl, 5% glycerol, 200 mM imidazole) in fractions. Eluted protein was loaded onto the Superdex S-200 gel filtration column equilibrated with buffer 25 mM Tris pH 7.5, 0.2 M NaCl. The collected fractions were analyzed on SDS-PAGE and pure fractions were pooled, dialyzed at 4 °C against dialysis buffer (25 mM Tris pH 7.5, 0.2 M NaCl, 5 mM DTT, 5% glycerol). The protein was concentrated to 8–10 mg/mL and further used for crystallization.

### Crystallization and structure determination

After initial screening, crystals were grown using the hanging drop vapor diffusion method by mixing 1 μL of protein (8 mg/mL) and 1 μL of reservoir solution containing 0.3 M Magnesium chloride hexahydrate, 18% PEG 3350, 10% glycerol. The obtained crystals were harvested using 20% glycerol as cryoprotectant and flash-frozen in liquid nitrogen. The dataset was collected at Beamline 30 A at the European Synchrotron Radiation Facility, Grenoble. The crystal diffracted to 2.4 Å resolution with space group P4_1_2_1_2. The data were indexed and scaled using XDS^29^. The StGyrA-CTD structure was solved by molecular replacement method using *E.coli* GyrA-CTD (PDB 1ZI0)^16^ as a template with the help of CCP4, PHASER^30^. Several cycles of manual model rebuilding and refinement were carried out using Coot^31^ and PHENIX^32^ respectively. In the structure, the region from residues 560 to 574 could not be resolved due to disorderliness. Likewise, side chains of residue Y557, E558 and Q560 were modeled as alanine due to lack of electron density. The final model refined to R_Cryst_ /R_free_ value of 19.0/24.0 with 97.9% residues in the most favored region and 2.1% in the additionally allowed region of Ramachandran plot. All the figures in the manuscript were generated using Pymol^33^.

### Electrophoretic-mobility shift assay

Gel retardation assay for StGyrA-CTD was performed with two different lengths of DNA sequences, a 37 bp and 162 bp strong Gyrase site (SGS) from pBR322 plasmid. It is known from literature that the minimum and maximum length of DNA required for DNA Gyrase to bind is 35bp^25^ and 240bp^27^ respectively. The minimum length of 37 bp DNA was chosen for experiments based on earlier studies^17,24^. The intermediate length of 162 bp from pBR322 has been reported to bind to GyrA-CTD. The 162 bp DNA contains a strong gyrase binding site in pBR322 plasmid^28^ and hence was considered for this study. The studies with the 37 bp DNA, aided in the determination of the DNA binding ability of GyrA-CTD protein. Whereas, the 162 bp DNA led to the estimation of the ability of GyrA-CTD to bind and wrap the DNA around its β-pinwheel. The 37 bp oligonucleotide^24^ 5’-TAA AGT CTA GAG ACA CGC ATA GTC AAT GAC GGA GTT A-3’ and 5’-TAA CTC CGT CAT TGA CTA TGC GTG TCT CTA GAC TTT A-3’ was obtained from Integrated DNA Technologies and dissolved in TE buffer (10 m MTris, pH 8.0, 1 mM EDTA). The 162 bp (SGS) was PCR amplified using pBR322 plasmid as a template (forward primer, 5’ -CAA GCC GTC GAC ACT GGT CCC GCC A-3’; reverse primer, 5’ -CGC GAG GGA TCC TTG AAG CTG-3’)^28^. The EMSA reaction mixture was performed with StGyrA-CTD and DNA in a molar ratio of 5:1 for 37 bp and 20:1 for 162 bp with the buffer containing 20 mM Tris–HCl (pH 7.5), 55 mMKCl, 5 mM DTT, 5% glycerol and 4 mM MgCl_2_ for 30 min at 37 °C. Samples were loaded into 8% native EMSA polyacrylamide gel and electrophoresis was performed in 0.5X TBE buffer (45 mM Tris; 45 mM boric acid; 1 mM EDTA) for 2–3 hrs at 4 °C and then stained with ETBR (0.7 µg/ml) for 30 minutes.

### Fluorescence binding assay

Binding of DNA to StGyrA-CTD was determined using fluorescence based binding with FAM labeled 37 bp oligonucleotides. The DNA concentration was kept constant (20 nM) and the protein was varied from 0 to 8000 nM in a buffer containing 20 mM Tris-HCl, pH 7.5, 70 mM KCl, 10% glycerol, and 1 mM MgCl_2_. The fluorescence measurements were taken and the data were fitted using binding equation Y = Bmax*X/(Kd+X) + NS*X,where X and Y represent added ligand and total binding respectively, B_max_ is the maximum binding, K_d_ equilibrium dissociation constant and N_s_ slope of the nonlinear regression progression.

### Protein – DNA Docking

Effect of mutations on GyrA-CTD - DNA complex interactions were observed by molecular docking. StGyrA-CTD protein and 37 bp DNA were docked using HADDOCK server^34^. The 37 bp DNA was modeled using CHIMERA^35^ and the basic residues exposed on the surface of the β-pinwheel of StGyrA-CTD were defined as active residues or interactive restraints of protein. The docking protocol initially involved orientation optimization followed by rigid body energy minimization and generated 1000 different complex orientations with minimum intermolecular energy function. Out of the 1000 orientations, the best 200 were refined and interface residues were subjected to simulated annealing. An interface residue includes the active and passive residues defined as the ambiguous interactive restraints (AIR). Simulated annealing allows conformational rearrangements to achieve the interface packing with improved energetics. The final step of explicit water refinement resulted in structures clustered using pairwise backbone root-mean-square deviation (r.m.s.d.) at the protein-DNA binding interface^36,37^. The clusters were evaluated and ranked based on average interactive energies including van der Waals, electrostatic and AIR energies. The cluster with the least intermolecular energy score was chosen as a final docked model.

## Supplementary information

Supplementary information.
