# Supplementary material for: The pivot point arginines identified in the β-pinwheel structure of C-terminal domain from Salmonella Typhi DNA Gyrase A subunit
Source: Sci Rep. 2020 May 8;10:7817. doi: 10.1038/s41598-020-64792-w (PMC7210945; doi:10.1038/s41598-020-64792-w)
Supplement: Supplementary file 1 — Supplementary information. [file 41598_2020_64792_MOESM1_ESM.docx]

**Supplementary Figures**

# Title:

# The pivot point arginines identified in the β-pinwheel structure of C-terminal domain from Salmonella Typhi DNA Gyrase A subunit

**Ekta Sachdeva, Gurpreet Kaur, Pragya Tiwari, Deepali Gupta, Tej P. Singh, Abdul S. Ethayathulla, Punit Kaur***

^1^Department of Biophysics, All India Institute of Medical Sciences, New Delhi 110029, India

*** Corresponding Author**

**Dr. Punit Kaur**

Professor and Head

Department of Biophysics

All India Institute of Medical Sciences

New Delhi -110029, India

E-mail: [punitkaur1@hotmail.com](mailto:punitkaur1@hotmail.com)

Tel: +91-11-2659 4288

Fax: +91-11-2658 8663

**
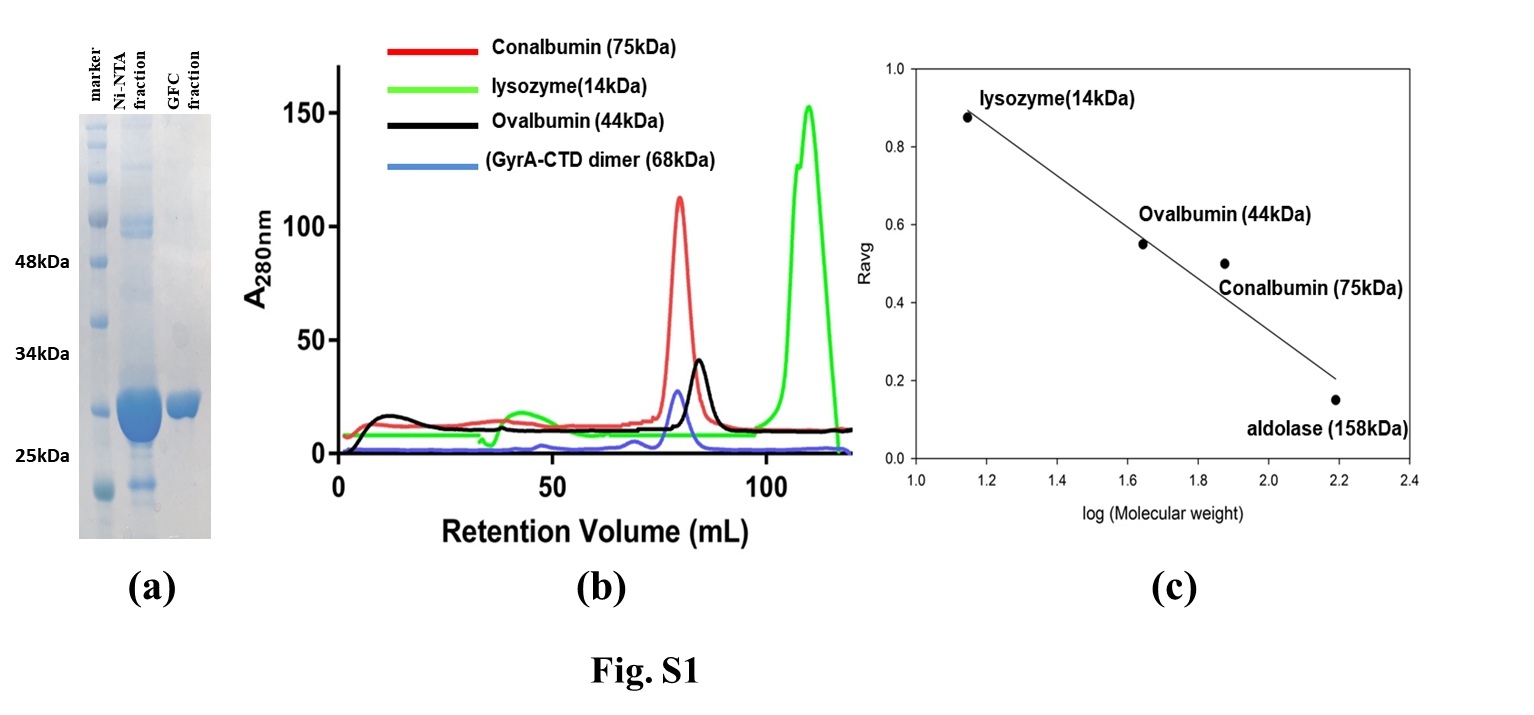
**

**Figure S1:** (**a**) 12% SDS gel analysis showing purified protein after Ni-NTA affinity chromatography and gel filteration chromatography. **(b**). Size exclusion chromatogram of StGyrA-CTD and standard proteins. **(c**)A plot of Ravg vs log (molecular weight) showing all standard proteins used -Conalbumin, Ovalbumin, Lysozyme, Aldolase


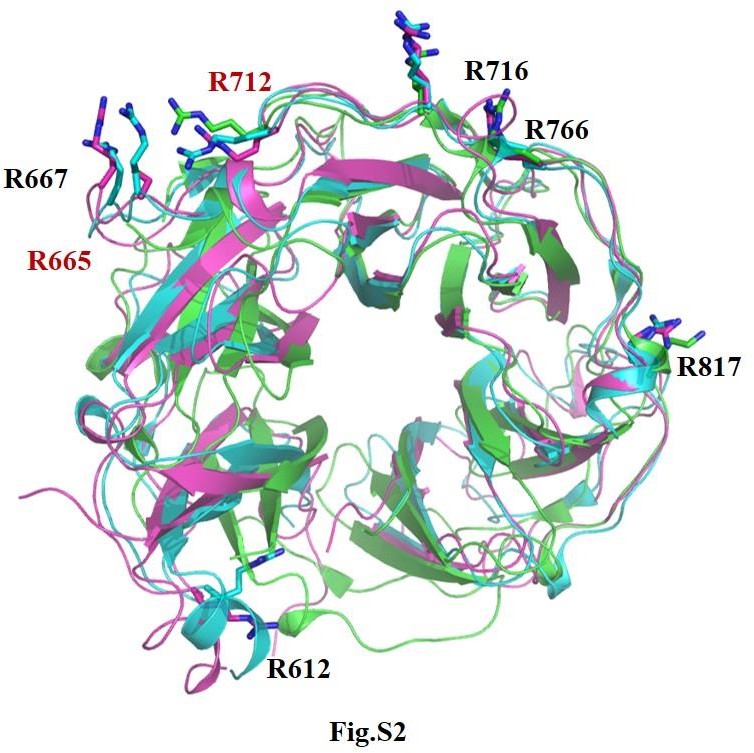


**Figure S2:** Superimposition of GyrA-CTD from different species showing conserved arginine residues selected from each blade. GyrA-CTD in magenta, cyan and green corresponds to S.Typhi, *M.tuberculosis* and *B.burgodorferi* respectively. Residues labeled in black are conserved arginine from each blade along with supporting residue in red.


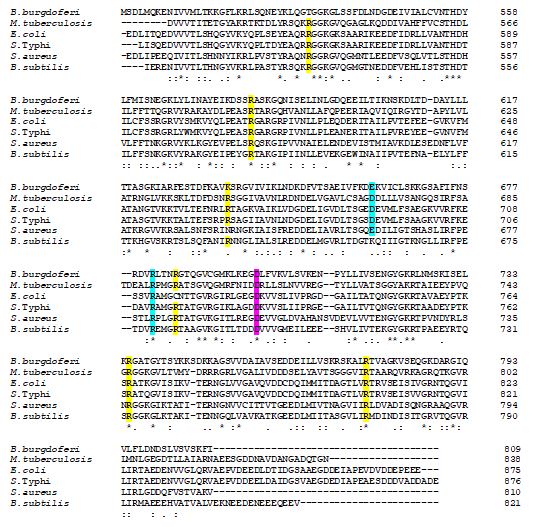


**Fig. S3**

**Figure S3:** Sequence alignment of GyrA-CTD from different bacterial species showing conserved residues selected for biochemical study. Residues highlighted in yellow are the conserved residues from each blade, residues in cyan are the supporting residue in blade3 and blade4, residues in magenta is the D692 residue selected from M.tb study.
